# Dual-Emission Fluorescence Probe Based on CdTe Quantum Dots and Rhodamine B for Visual Detection of Mercury and Its Logic Gate Behavior

**DOI:** 10.3390/mi12060713

**Published:** 2021-06-18

**Authors:** Yuefeng Gao, Sai Xu, Zhijian Liu, Kezhen Yu, Xinxiang Pan

**Affiliations:** 1 College of Marine Engineering, Dalian Maritime University, Dalian 116026, China; gaoyuefeng@dlmu.edu.cn (Y.G.); liuzhijian@dlmu.edu.cn (Z.L.); yukezhen@dlmu.edu.cn (K.Y.); 2 School of Science, Dalian Maritime University, Dalian 116026, China; 3 Maritime College, Guangdong Ocean University, Zhanjiang 524088, China

**Keywords:** CdTe quantum dots, fluorescent nanomaterials, dual-emission fluorescence probe, mercury ions detection, IMPLICATION logic gate

## Abstract

It is urgent that a convenient and sensitive technique of detecting Hg^2+^ be developed because of its toxicity. Conventional fluorescence analysis works with a single fluorescence probe, and it often suffers from signal fluctuations which are influenced by external factors. In this research, a novel dual-emission probe assembled through utilizing CdTe quantum dots (QDs) and rhodamine B was designed to detect Hg^2+^ visually. Only the emission of CdTe QDs was quenched after adding Hg^2+^ in the dual-emission probe, which caused an intensity ratio change of the two different emission wavelengths and hence facilitated the visual detection of Hg^2+^. Compared to single emission QDs-based probe, a better linear relationship was shown between the variation of fluorescence intensity and the concentration of Hg^2+^, and the limit of detection (LOD) was found to be11.4 nM in the range of 0–2.6 μM. Interestingly, the intensity of the probe containing Hg^2+^ could be recovered in presence of glutathione (GSH) due to the stronger binding affinity of Hg^2+^ towards GSH than that towards CdTe QDs. Based on this phenomenon, an IMPLICATION logic gate using Hg^2+^/GSH as inputs and the fluorescence signal of QDs as an output was constructed.

## 1. Introduction

Mercury, a famous chemical pollutant, is a great hazard to public health, as even trace amounts of mercury in the blood can poison the central nervous and endocrine systems [1,2]. Moreover, mercury cannot be decomposed once it is released into the environment, it can be absorbed and then stay inside the bodies of animals or plants to contaminate them, and when these are food products, such as vegetables, fruit, rice and even dairy products, finally it will be enriched in the human body through the food chain [3,4]. Therefore, it is critical to develop sensitive probes which can detect trace mercury in biological and environmental samples. Up to now, many techniques have been developed to determine mercury ions, including inductively coupled plasma mass spectrometry, atomic absorption spectrometry, anodic stripping voltammetry and electrochemical methods, etc. [5,6,7,8]. These analytical techniques can measure the levels of mercury sensitively and accurately, but they have to be operated using expensive and sophisticated equipment and require complicated sample preparation procedures, which limits their application for rapid and simple detection [9]. Therefore, a facile, rapid, inexpensive and highly sensitive technique to detect mercury ions is still needed.

Toward this goal, in recent years, much effort has been focused on developing novel fluorescent detection systems for mercury, including sensors based on gold nanoparticles, organic dyes, quantum dots (QDs) and lanthanide-doped nanoparticles, etc. [10,11,12,13,14]. Among them, QDs fascinate researchers and reveal promising prospects in the fluorescence detection field due to their distinctive photophysical properties, such as high quantum efficiency, size-dependent photoluminescence, broad excitation window, high extinction coefficients and tunable emission spectra [13]. The utilization of QDs as fluorescence labels in the analytical industry has been reported widely [15,16,17,18]. Most QD-based labels employ a single optical output for the detection. However, the change of emission intensity of a single emission band as a major sensing signal is easily disturbed by the experimental conditions, such as concentration of the probe, fluctuations of the source intensity, and the condition of the collection instrument. In contrast, the probes based on dual-emission materials can significantly improve the quantification accuracy because they are able to calibrate themselves via calculating two emission intensities instead of single intensity of one peak [19]. In general, dual-emission probes are built with two independent materials which possess different fluorescence emission wavelengths. The two emissions play different roles in analysis, as the emission peak from one of them can be used as reference signal, while the other can be used as a specific identification signal for the target analyte [20,21]. The variation of the ratio of fluorescence intensity between these two emissions leads to an obvious change of the fluorescence color. Thus, compared with the single emission intensity-based probe, the dual-emission probe is easier to detect by the naked eye. Among the research on dual-emission probes, there are many studies on the application of QD-based ratiometric fluorescent probes to metal ion detection, even including metal-free carbon QDs and graphene QDs [22,23,24].

In this study, aiming to explore the operability of QDs used as an optical signal probe, a novel probe using the dual-emission pathway was designed to detect mercury. The dual-emission probe employed CdTe QDs as sensing fluorophore and rhodamine B as the internal standard. There are three reasons for selecting rhodamine B as internal standard: first, the wavelength that excites rhodamine B and CdTe QDs is the same; second, the emission spectra of rhodamine B and CdTe QDs don’t overlap; third, rhodamine B isn’t sensitive to Hg^2+^. Thus, by adding a Hg^2+^ solution, due to the sensitivity of CdTe QDs to Hg^2+^, the green fluorescence was gradually reduced until invisible. Different from CdTe QDs, the red orange emission of rhodamine B was almost unchanged. Thus, the variation of Hg^2+^ concentration changed the emission intensity ratio of QDs and rhodamine B and simultaneously resulted in the color change of the probe, which contributed to detecting Hg^2+^ visually. Experimental results showed that the dual-emission probe has low detection limit, a good linear relationship as well as selectivity so that it has been successfully applied in drinking water, sea water and wastewater to detect Hg^2+^.

On the other hand, Hg^2+^ has very high affinity for the biomolecules containing thiol groups, such as GSH, Hcy, Cys and albumin. The formation constant for the binding of Hg^2+^ to thiol is about 10 orders of magnitude larger than that for the binding of Hg^2+^ to other nucleophiles if tested under the same conditions [25]. In consequence, upon adding thiol-containing biomolecules, the fluorescence quenched by Hg^2+^ recovered because of the strong preference for Hg^2+^ to bind biothiols. On the basis of turn on/off property of the probe, an implication logic gate using Hg^2+^/GSH as inputs and the fluorescence signal of emission of QDs as an output was designed.

## 2. Experimental Sections

### 2.1. Synthesis of CdTe QDs

Water-soluble CdTe QDs were prepared according to a previously described literature method [26]. Briefly, CdCl_2_·2.5H_2_O (0.04 M, 16 mL) was diluted in 200 mL deionized water in a one-necked flask, then Na_2_TeO_3_ (0.01 M, 4 mL), trisodium citrate dehydrate (400 method), NaBH_4_ (400 mg) and mercaptosuccinic acid (MSA, 200 mg) were added to the solution simultaneously with stirring. After the color of the solution turned green, the flask was attached to a condenser to reflux for 40 min under an open-air environment. The CdTe QD product was washed using ethanol and isolated by centrifugation. Finally, the CdTe QDs product was dispersed in water for further use.

### 2.2. Detecting of Mercury in Aqueous Solution

To fabricate the dual-emission sensor, 2 mL of CdTe QDs (6 mM) and 20 μL rhodamine B (0.03 mM) were blended in Tris-HCl buffer (pH = 7.5) and ultrasonically agitated for 20 min. For Hg^2+^ detection, 20 μL solutions containing different levels of Hg^2+^ were added into the CdTe QDs and rhodamine B mixture with stirring. After allowing the mixture to react for 5 min, the mixture solution was illuminated with UV light to study the color changes. To determine the specificity of the dual-emission probe for Hg^2+^ ion, various metal ions such as Pb^2+^, K^+^, Na^+^, Ca^2+^, Mg^2+^, Zn^2+^, Cd^2+^, Ag^+^, Ni^2+^ and Co^2+^ with a concentration of 500 nM were prepared. Then these solutions and Hg^2+^ solution, also with the concentration of 500 nM, were added to the probe individually and the alterations of fluorescence were recorded. To demonstrate the practicability of this kind of dual-emission probe, Hg^2+^ analysis in drinking water, sea water and wastewater was investigated. Detection in deionized water was conducted as a control. With a specific concentration, CdTe QDs were mixed with rhodamine B in four kinds of water and ultrasonically agitated for 20 min. Then given amounts of Hg^2+^ at different concentrations were introduced into the mixture with stirring simultaneously. Five min later, the fluorescence spectrum was measured and the fluorescence intensity (spectral integral intensity) changes were calculated. The concentrations of Hg^2+^ in actual samples were quantified by comparing the corresponding intensity changes with the linear plot obtained in Tris-HCl buffer.

### 2.3. Characterization

High-resolution transmission electron microscope (HR-TEM) images of as-prepared CdTe QDs were recorded by on a Tecnai G2 S-Twin microscope (FEI, Hillsboro, OR, USA) which was manipulated with an acceleration voltage of 200 kV. The phase structure of the samples was characterized via X-ray diffraction (D/max-rA power diffractometer (Rigaku, Tokyo, Japan) using Cu-KR radiation, λ = 1.54178 Å). Fluorescence spectra were acquired via an F-4600 spectrophotometer (Hitachi, Tokyo, Japan). The detection solution was placed in a quartz cuvette with 2 mL volume and 1 cm light path. And the probe was excited with the 365 nm wavelength. All the assays were operated at room temperature. All error bars express standard deviations from experiment which was repeated three times under same condition.

## 3. Results and Discussion

### 3.1. Morphology and Structure of CdTe QDs

**Figure 1:**
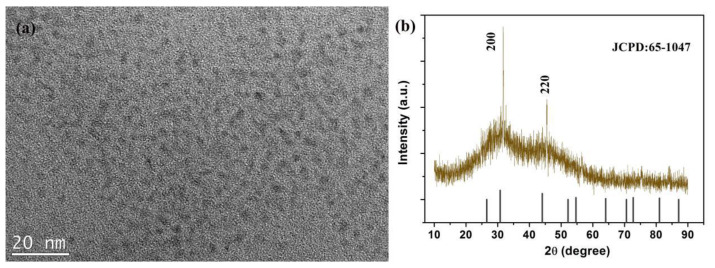
(**a**) HR-TEM image of CdTe QDs. (**b**) XRD patterns of the CdTe QDs.

Without being modified the surface further, CdTe QDs were still able to disperse well in aqueous medium, which led to the formation of steady colloidal solutions directly. Figure 1a exhibits the HR-TEM picture of CdTe QDs dispersing in water, in which the size of the CdTe QDs product is evaluated as ~2.1 nm, the size of QDs can be computed based on its ultraviolet-visible absorption spectra. Peng et al., generalized an experience formula to calculate the size of the CdTe QDs based on a large number of experimental results [27]:

(1) $$D=\left(9.8127\times 10^{-7}\right)\lambda^{3}-\left(1.7147\times 10^{-3}\right)\lambda^{2}+1.0064\lambda -194.84$$

where *D* (nm) represents the particle diameter of the QDs, and *λ* (nm) represents the wavelength of absorption peak. (The absorption spectrum of QDs was shown in Figure S1) With the utilization of the formula above, the mean diameter of QDs was quantified to be 1.79 nm, approximately equivalent to the HR-TEM image. Figure 1b exhibits X-Ray diffraction patterns of CdTe QDs, which shows all the diffraction peaks of the CdTe QDs can be indexed to the cubic structure of CdTe QDs phase in accordance with JCPDS file 65-1047, indicating that the product is pure in phase.

### 3.2. Luminescence Characterization of the Dual-Emission Probe

**Figure 2:**
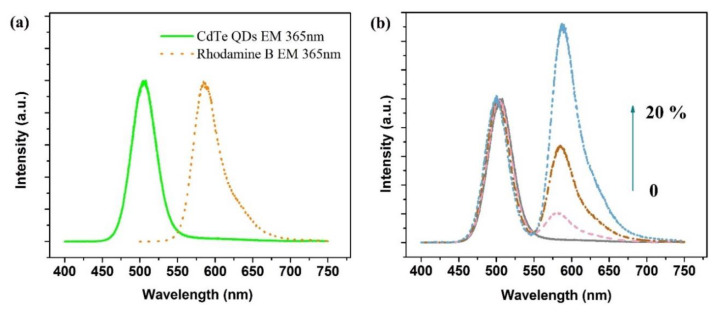
(**a**) Emission spectra of the QDs (green solid line) and rhodamine B (orange dashed line) excited at 365 nm. (**b**) Emission spectra of the mixed solution containing different rhodamine B concentrations under excitation of 365 nm.

Figure 2a shows the emission spectra of the QDs (green solid line) and rhodamine B (orange dashed line) excited at 365 nm. As for QDs, the emission band locates at 510 nm, which originates from the radiation resulting from recombination of electrons and holes that are trapped in surface defect states, while the emission band of rhodamine B in the range of 550 nm–700 nm is centered at 585 nm, sharing nothing in common with the emission band range of the QDs. To evaluate whether there is energy transfer between CdTe QDs and rhodamine B, different amount of rhodamine B solution was mixed into the QDs. Figure 2b shows the spectra of the mixed solution excited at 365 nm. It shows that the emission band of rhodamine B gradually rises with the concentration of rhodamine B addition from 0 to 20%, while the emission intensity of QDs is almost unchanged. It can be concluded that there is no energy transfer between CdTe QDs and rhodamine B. The luminescence independence of the two fluorophores ensures the accuracy of this kind of dual-emission probe. To figure out the binding form of QDs and rhodamine B in the mixed solution, the ζ potential of the two materials and the mixture were measured. From the measurement, QDs demonstrated negative charge (ζ potential = −15.37 mV), rhodamine B had positive charge (ζ potential = +3.78 mV) and the mixture had negative charge (ζ potential = −6.24 mV). The converse potential results in strong electrostatic interaction between rhodamine B and QDs. Thus, the rhodamine B molecule was considered to have attached to the surface of the QDs to form the dual emission probe.

### 3.3. Fluorescence Response Characteristics of Dual-Emission Probe for Detecting Hg^2+^

**Scheme 1:**
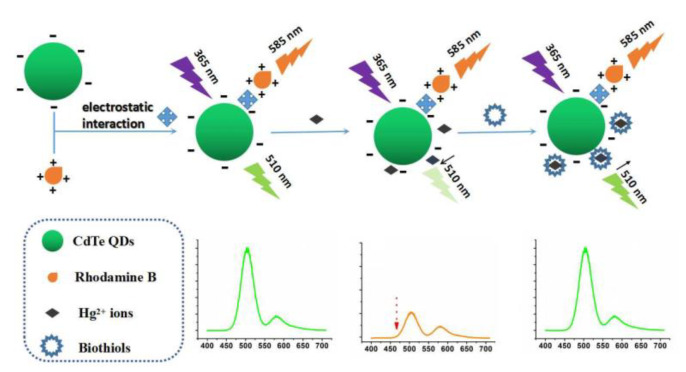
Principle of Hg^2+^ detection by a dual-emission probe and the florescence recovery by biothiols.

**Figure 3:**
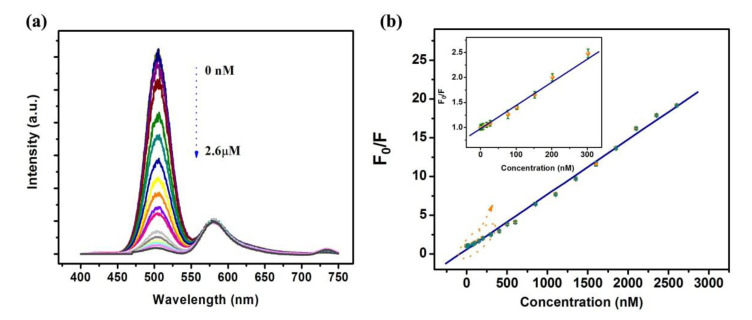
(**a**) Emission spectra of dual-emission probes with addition various Hg^2+^ concentrations excited at 365 nm. (**b**) Stern-Volmer plot of the fluorescence of probes versus concentration of Hg^2+^ ions.

The principle of mercury detection using a dual-emission probe and the fluorescence recovery by biothiols is illustrated in Scheme 1. After simply mixing QDs and rhodamine B, rhodamine B molecules attach to the surface of the QDs via electrostatic interaction, the mixture solution displays two kinds of emission peaks centered at 510 nm and 585 nm upon excitation at 365 nm, corresponding to the photoluminescence of CdTe QDs and rhodamine B, respectively. After adding mercury to the mixture, the green emission of the QDs decreases, meanwhile, the emission of rhodamine B maintains a relatively constant intensity. As rhodamine B is insensitive to Hg^2+^ (Figure S2), the introducing of Hg^2+^ merely changes the emission intensity of QDs, so each spectrum is normalized with the emission intensity of rhodamine B. The normalized emission spectra of the dual-emission probe versus the concentration of Hg^2+^ excited at 365 nm are exhibited in Figure 3a. It shows that the green emission of the QDs gradually decreases in pace with the concentration of Hg^2+^, which was added into the mixed solution, increasing from 0 to 2.6 μM, but without a peak shift. Figure 3b shows the level of fluorescence quenching of QDs relies on the concentration of Hg^2+^, which is best described using the Stern–Volmer equation [28]:

F_0_/F = K_sv_[Q] + 1 (2)

where F_0_ and F are the fluorescence intensities of QDs with Hg^2+^ in absence and at presence, respectively, [Q] represents the concentration of Hg^2+^, and K_sv_ represents the Stern–Volmer constant. The signal of F_0_/F increases stably with the concentration of Hg^2+^ increasing from 0 to 2.6 μM. A good linear relationship exists between fluorescent intensity ratio and the concentration of Hg^2+^. The correlation coefficient of the linear fit, represented as R, is 0.998. The LOD of the dual-emission probe was estimated from the formula of 3σ/S and found to be 11.4 nM, where σ stands for the standard deviation of the blank signal and S represents the slope of the calibration plot [29]. In comparison with other methods of detecting Hg^2+^ reported previously, the proposed method exhibits pretty good linear range and detection limit, as listed in Table 1 [30,31,32,33,34]. Furthermore, the relative standard deviation (RSD) for the tests, measuring 1 μM Hg^2+^ independently three times, is 1.32%.

**Figure 4:**
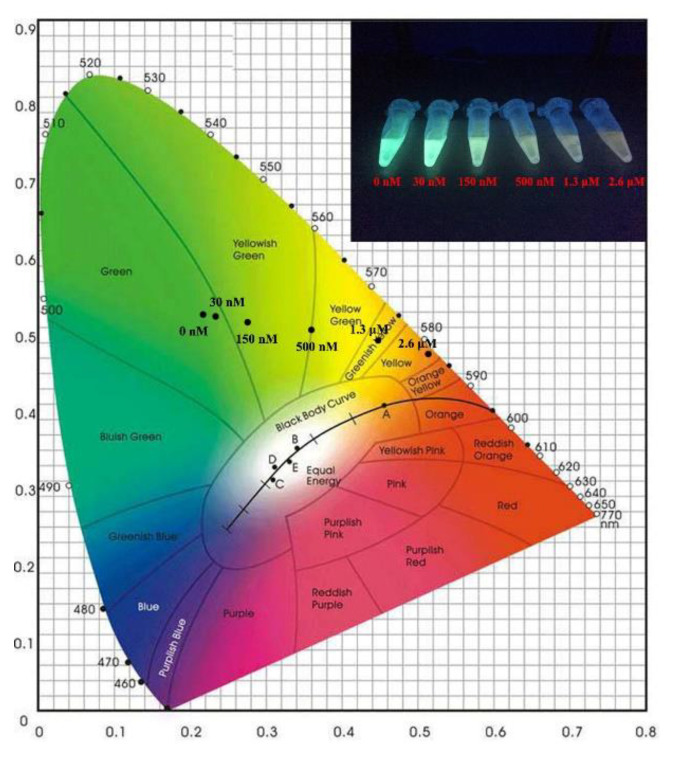
CIE chromaticity coordinates for dual-emission probes with various concentrations of Hg^2+^. The inset exhibits the corresponding fluorescence images, all photos were taken using a UV lamp.

It should be noted that the changing photoluminescence intensity ratio can engender a color change visible to the naked eye, which enables the dual-emission probe to detect Hg^2+^ visually. As shown the inset of Figure 4, the color of the dual-emission probes changes from green to yellow gradually in pace with the addition of Hg^2+^, through which the naked-eye is able to detect Hg^2+^. Moreover, the diagram of CIE chromaticity is expressed by the nominal value, which x is the red component and y is the green component, and the figures on the border suggest the wavelength of the spectrum. All the actual colors existing in Nature are located inside the range of the closed curve. The CIE chromaticity coordinates for dual-emission probe with different levels of Hg^2+^ are exhibited in Figure 4, the emission light changes its color from green to yellow gradually in pace with the concentration of the CEA increasing from 0 to 2.6 μM. The result of the CIE chromaticity is accordant with the naked eye detection.

### 3.4. Fluorescence Response Characteristics of QD-Based Probe for Hg^2+^ Detection

**Figure 5:**
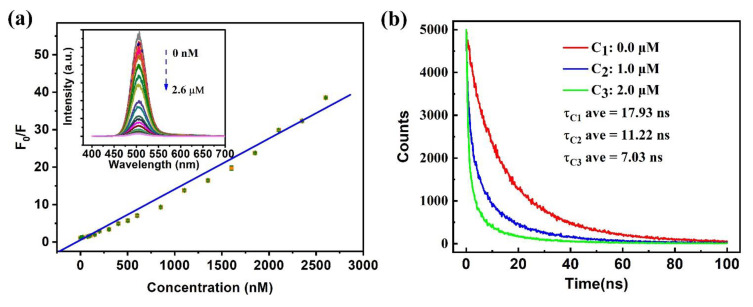
(**a**) Stern-Volmer plot of the fluorescence of QD-based probes versus the level of Hg^2+^. The inset shows the emission spectra of QD-based probes with addition various Hg^2+^ concentrations under excited at 365 nm. (**b**) Time-resolved PL decay curves of CdTe with different Hg^2+^ concentration.

To demonstrate the advantage of the dual-emission probe, the fluorescence response of the QD-based probe to the addition of different amounts of mercury were investigated under the same experimental conditions. The fluorescence intensities of QD-based probe are continuously reduced with the increasing concentration of Hg^2+^, as exhibited in the inset of Figure 5a. The calibration plot of F_0_/F versus the concentration of Hg^2+^ shows a linear relationship within the range of 0–2.6 μM. R, the correlation coefficient used for the linear fit, is 0.986. The LOD of the QD-based probe is found to be 24.9 nM. Compared with the probe based on single wavelength intensity, the probe with double emissions has better linear relationship, lower LOD, and is easier to detect with the naked eye.

As shown in the inset of Figure 5a, the emission intensity of CdTe QDs reduces gradually with the increasing concentration of mercury ions without any obvious changes in the maximum emission wavelength and spectral width. Previous publications reported that Ag^+^ could quench the fluorescence of 3-mercaptopropionic acid (MPA)-capped CdTe QDs. However, different from Hg^2+^, the emission spectra of CdTe QDs quenched by Ag^+^ ions exhibit an obvious red shift accompanied by the changes in the spectra profiles. As discussed in the former reports, the mechanism for the CdTe QD fluorescence quenching was that the binding of Ag^+^ with bare Te atoms could form a Ag_2_Te species on the surface of the QDs and thus alter the surface state of QDs [35]. As for Hg^2+^, the quenching mechanism is complex: electron transfer as well as ion-binding can result in the reduction of QD emission intensity. Because of the strong affinity for the surface ligands of CdTe QDs, Hg^2+^ can bind to the surface of QDs, but the primary role for Hg^2+^ to quench the fluorescence of QDs is electron transfer, which is in accord with the fact that no spectraal shift found in the quenching process [36]. Recombination of charge carriers immobilized in traps of various energies leads to the broad photoluminescence emission. During the course of fluorescence quenching of CdTe QDs, various energy traps recompose via electron transfer from surface traps of CdTe QDs to Hg^2+^, as described below [37,38]:

CdTe + *hν*_ex_ → CdTe (e^−^/h^+^) → CdTe (e_tr_^−^/h_tr_^+^)

CdTe (e_tr_^−^/h_tr_^+^) → CdTe + *hν*_em_

CdTe (e_tr_^−^/h_tr_^+^) + Hg^2+^ → CdTe (h_tr_^+^) + (Hg^2+^ ● e-)* → CdTe + Hg^2+^ + heat

To verify the conjecture, the CdTe QDs time-resolved PL decay curves of multiple mercury ions contents were measured. As shown in Figure 5b, the curves were fitted to obtain the average fluorescence decay lifetime. As revealed from the results, with the increase in the content of Hg^2+^, the decay lifetime of CdTe QDs gradually decreased, reflecting a significant electron transfer from CdTe QDs to Hg^2+^. The increase in the quencher Hg^2+^ content caused the excitons to decay faster, thereby reducing the fluorescence decay lifetime. As reported before [39], the PL decrease is caused mostly by either dynamic quenching featuring decreased PL lifetime or static quenching typical of shifted emission and/or absorption spectra. In this case, the unshifted emission peak and the decreased decay lifetime of QDs with the increase of Hg^2+^ concentration indicate that dynamic quenching is the main factor leading to the observed fluorescence quenching.

### 3.5. Selectivity to Metal Ions and Time Effect to Hg^2+^ of Dual-Emission Probe

**Figure 6:**
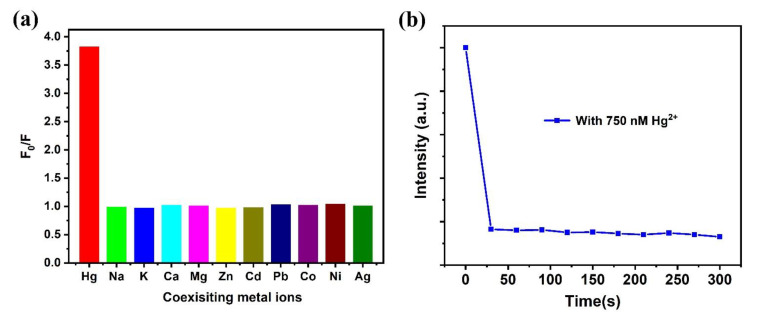
(**a**) Detection specificity for Hg^2+^ (the concentrations of all ions are 500 nM). (**b**) Time effect of the dual-emission probe for mercury detection.

To estimate the selectivity of the dual-emission probe for mercury detection, different metal ions were tested along with Hg^2+^. As exhibited in Figure 6a, only Hg^2+^ results in a large variation of the fluorescence intensity, as other ions don’t show any obvious fluorescence intensity variation to the dual-emission probe. Selectivity coefficients, used for the probe response ratio generated from interfering ions and target ions are listed in Table S1. Interfering ions exhibit the selectivity coefficients located within the range of 0.0013–0.0121. The results show the great specificity of the dual-emission probe for Hg^2+^ ions. Furthermore, the time effect of dual-emission probe for mercury ions detection was also investigated, as shown in Figure 6b. After adding 750 nM Hg^2+^ ions, the luminescence intensity reduces quickly in less than 30 s, then stays stably in 5 min. These results show that the reaction of Hg^2+^ with CdTe QDs is very quick and the luminescence is stable in a long time, which are in favor of practical application.

### 3.6. Detection in Real Samples

To evaluate the practicality of the dual-emission probe, the detection of Hg^2+^ in drinking water, sea water and wastewater were investigated. The drinking water was three-stage filtered water from the Luminescence and Optoelectronic Technology Laboratory of Dalian Maritime University, and the sea water was obtained from Dalian port. The wastewater obtained from local company simply underwent centrifugation to remove large aggregated particles to prepare a Hg^2+^ stock solution. In addition, the detection of Hg^2+^ in deionized water was also added as a control. Upon adding different concentrations of Hg^2+^ solution, a visible and obvious color change was observed. The corresponding intensity changes were compared with the standard plot investigated in Tris-HCl buffer to assess the effectiveness of the dual-emission probe for detecting Hg^2+^ in actual samples, as summarized in Table 2. It can be seen that the concentration of Hg^2+^ obtained from the standard plot is in pretty good accord with that added and ICP-MS results. This completely proves the applicability in real water samples of the probe with double emissions to detect Hg^2+^ visually.

### 3.7. IMPLICATION Logic Gate

**Figure 7:**
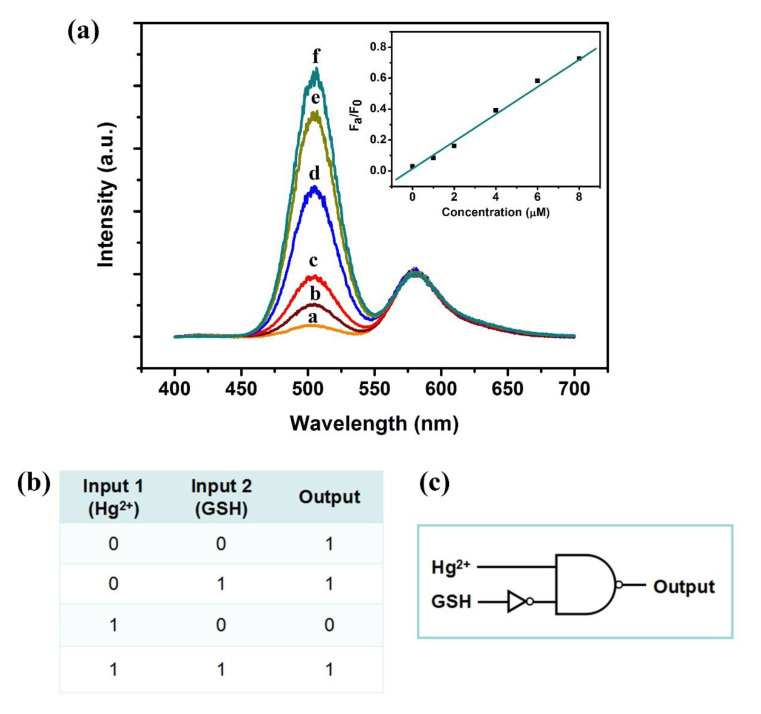
(**a**) Fluorescence recovery spectra of probe/Hg^2+^ system with varying concentration of GSH (from b to f: 1, 2, 4, 6, 8 μM), curve a is the emission spectrum of probe with 2 μM Hg^2+^, inset: the relationship between F_a_/F_0_ and GSH concentration. F_0_ is fluorescence intensity of probe ranging from 450 nm to 550 nm, and the F_a_ is the fluorescence intensity recovered due to presence of GSH, respectively. Truth table (**b**) and symbol (**c**) of the IMPLECATION gate.

As shown in Scheme 1, in the presence of Hg^2+^, the fluorescence of QDs is faint because of the quenching performance of Hg^2+^, however, adding biothiols to the QDs/rhodamine B-Hg^2+^ system, the fluorescence of QDs is regained due to the priority of Hg^2+^ to bind to biothiols. Here, glutathione (GSH), a common used biothiol, was selected as fluorescence recovery agent. To explore the recovery effect of GSH, various amounts of GSH were added to the dual-emission probe with 2 μM Hg^2+^. As exhibited in Figure 7a, the fluorescence intensity is obviously recovered when 1 μM GSH is present in the mixture solution, and it increases gradually in pace with the concentration of GSH increasing (from b to f in Figure 7a). The inset of Figure 7a reveals the relationship of the fluorescence intensity recovery of dual-emission probe ranging from 450 nm to 550 nm versus the concentration of GSH. It showed good linear relationship inside the range of 0–8 μM, and the intensity recovered to 80% of the original intensity by adding 8 μM GSH.

Based on the Hg^2+^ and GSH-sensitive switch system, an IMPLICATION logic gate using Hg^2+^ and GSH as input, and the fluorescence signal of dual-emission probe ranging from 450 nm to 550 nm as output was constructed. For input, the presence of Hg^2+^ or GSH was set as “1”, and the absence of those as “0”. Only with the input of Hg^2+^ and without GSH (1/0), the fluorescence intensity of the dual-emission ranging from 450 nm to 550 nm was low, and the output was “0”. The outputs were all “1” in other cases. The truth table and symbol of the IMPLICATION logic gate are shown in Figure 7b,c.

## 4. Conclusions

In summary, a fluorescence probe with double emission was designed to detect Hg^2+^ visually, which was constructed with the utilization of CdTe QDs (green fluorescence color) and rhodamine B (red orange fluorescence color. Compared to the probe based on single emission QDs, a better linear relationship between the variation of fluorescence intensity and the concentration of Hg^2+^ exists in the dual-emission probe, and the LOD is as low as 11.4 nM. In addition, the probe exhibits good anti-interference properties and has been successfully employed to detect the content of Hg^2+^ in drinking water, sea water and wastewater samples, suggesting its great practicability for application in visual detection of mercury in diverse analytical fields. Moreover, due to the stronger binding affinity of Hg^2+^ towards GSH than that towards CdTe QDs, adding GSH to the dual-emission probe/Hg^2+^ solution leads to the recovery of QDs emission, thus, an IMPLICATION logic gate using Hg^2+^/GSH as inputs and the fluorescence signal of emission of QDs as an output was designed to simulate this experiment.

## Figures and Tables

**Table 1:** Detection limit of Hg^2+^ and linear ranges of six kinds of fluorescence probes.

Probes	Linear Range (nM)	LOD (nM)	Ref.
C-dots	0–25,000	23.4	[30]
N, S-MoSe_2_	0–5000	10	[31]
Thioctic acid-carbon dots	50–580	33.3	[32]
DNA-templated AgNCs	0–2000	5	[33]
Nitrogen-doped C-dots	300–5000	24.1	[34]
CdTe QDs	0–2600	11.4	This work

**Table 2:** Utilization of dual-emission probe to detect Hg^2+^ in actual samples.

Samples	-	By Our Method		By ICP-MS	
	Added (nM)	Measured (nM)	Recovery (%)	Measured (nM)	Recovery (%)
Drinking water	100.0	97.5	97.5	98.9	98.9
	500.0	512.8	102.6	510.7	102.1
	2000.0	2025.8	101.3	2026.5	101.3
Sea Water	100.0	103.7	103.7	103.9	103.9
	500.0	523.9	104.8	527.5	105.5
	2000.0	2043.8	102.2	2028.7	101.4
Wastewater	100.0	110.6	110.6	108.4	108.4
	500.0	530.5	106.1	527.8	105.6
	2000.0	2065.4	103.3	2041.8	102.1
Deionized Water	100.0	97.5	97.5	98.6	98.6
	500.0	517.6	103.5	505.3	101.1
	2000.0	2037.9	101.9	2020.5	101.0

## Supplementary Materials

The following are available online at https://www.mdpi.com/article/10.3390/mi12060713/s1, Figure S1: Absorption spectrum of CdTe QDs, Figure S2: Emission spectra of Rhodamine B with different concentrations of Hg^2+^ under excitation of 365 nm, Table S1: Selectivity coefficients of the interference ions.
