# Supplementary material for: Dual-Emission Fluorescence Probe Based on CdTe Quantum Dots and Rhodamine B for Visual Detection of Mercury and Its Logic Gate Behavior
Source: Micromachines (Basel). 2021 Jun 18;12(6):713. doi: 10.3390/mi12060713 (PMC8234752; doi:10.3390/mi12060713)
Supplement: Supplementary file 1 [file micromachines-12-00713-s001.zip › micromachines-1249376-SI.pdf]

Supplementary: Dual-emission fluorescence probe based on CdTe Quantum Dots and Rhodamine B for visual detection of mercury and its logic gate behavior

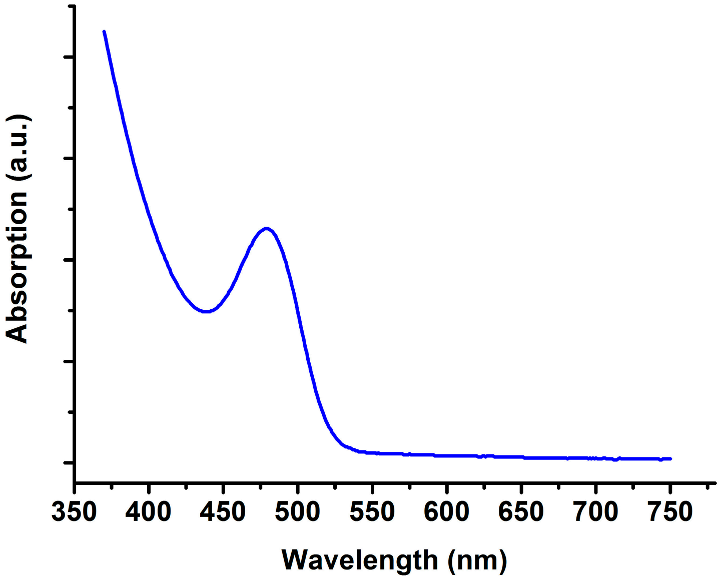

Figure S1. Absorption spectrum of CdTe QDs.

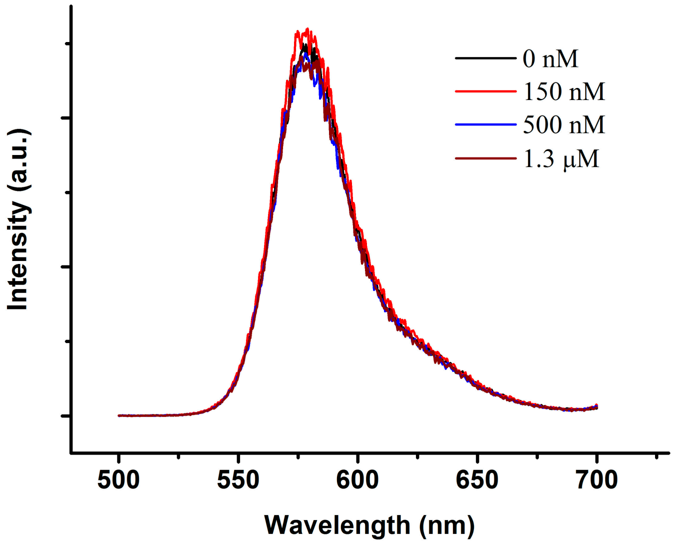

Figure S2. Emission spectra of Rhodamine B with different concentrations of Hg^{2+} under excitation of 365 nm.

Table S1. Selectivity coefficients of the interference ions.

**Ions.**	**Na^{+}**	**K^{+}**	**Ca^{2+}**	**Mg^{2+}**	**Zn^{2+}**	**Cd^{2+}**	**Pb^{2+}**	**Co^{2+}**	**Ni^{2+}**	**Ag^{+}**
Selectivity coefficients	0.0013	0.0058	0.0073	0.0042	0.0060	0.0037	0.0092	0.0063	0.0121	0.0047
